# The emperor has no clothes: a synthesis of findings from the Transformative Research on the Alcohol industry, Policy and Science research programme

**DOI:** 10.1111/add.16058

**Published:** 2022-10-24

**Authors:** Jim McCambridge, Gemma Mitchell, Matthew Lesch, Andreas Filippou, Su Golder, Jack Garry, Andrew Bartlett, Mary Madden

**Affiliations:** ^1^ Department of Health Sciences University of York Heslington York UK

**Keywords:** Alcohol industry, commercial determinants of health, corporate power, policy, public health, science

## Abstract

**Background and Aims:**

The Transformative Research on the Alcohol industry, Policy and Science (TRAPS) programme investigates the alcohol industry, with an innovative focus on public health sciences. TRAPS adds to an under‐developed literature on the study of alcohol industry influence on alcohol science and policymaking. This paper provides a synthesis of TRAPS findings to inform future research.

**Methods:**

We conducted an interpretive review of TRAPS research findings across its component studies, identifying and integrating the key contributions made by individual studies to the literature on alcohol policymaking and science, and identifying areas where TRAPS progress was limited. This produced themes for consideration in future research agenda setting.

**Results:**

TRAPS explored the interventions of the alcohol industry in science and policymaking using various methods, including systematic reviews and qualitative interviews. These studies identified the industry’s activities in several key areas, such as the debate over minimum unit pricing (MUP), cardiovascular health and alcohol research and a long‐running public relations programme developed in close connection with the tobacco industry. Collectively, the research shows that alcohol policymaking has involved a contest between the research community and alcohol industry actors about whether and how science should be used to inform policy.

**Conclusions:**

The TRAPS programme demonstrates the need for a transdisciplinary approach to understand the nature of corporate political activity; the crucial role industry involvement in science plays in the development of corporate political power; and how public health actors have successfully overcome industry opposition to evidence‐based policies. Advances in alcohol policy should be underpinned by strong, reflexive public health sciences, alert to the role of industry in the alcohol harms under study and thorough in their investigation of the alcohol industry as an object of study in itself.

## INTRODUCTION

Alcohol is responsible for approximately 3 million deaths globally every year [1]. The Transformative Research on the Alcohol industry, Policy and Science (TRAPS) research programme was established in 2016 to investigate the alcohol industry and public health sciences and policy. TRAPS had its origins in an earlier set of studies that examined alcohol industry influence on British alcohol policy [2, 3, 4]. There had previously been little study of alcohol industry methods of influencing public policies in Britain [5, 6] or elsewhere [7, 8, 9, 10, 11].

Baggott [6] made the arresting observation that in Britain, as elsewhere, national alcohol policies typically operated directly in opposition to the research evidence; ineffective policies were the norm, and scientific evidence was largely ignored in policymaking. Alcohol industry actors were highly involved in British policymaking, building upon deep historical connections to the Conservative Party [6], also extending to the Labour Government from 1997 to 2010 [12]. With devolution of the hitherto highly centralized UK state, innovations in alcohol policy emerged in Scotland [4, 13]. In 2008, for example, the Scottish government initiated a consultation on a new alcohol strategy and invited submissions from stakeholders, including the alcohol industry. Subsequent analysis of industry submissions showed that alcohol industry actors misused scientific evidence in highly coordinated and multi‐faceted efforts to prevent the adoption of policy measures that ran counter to business interests [14].

This latter finding provided the specific stimulus for TRAPS. A preparatory study was undertaken on whether alcohol industry funding biased scientific data on alcohol’s purported cardiovascular benefits, identifying evidence of possible industry influence of findings on stroke and underscoring the need for rigorous research [15]. Other preparatory studies identified the multiplicity of actors involved, including so‐called ‘social aspects organizations’ [SAOs; corporate social responsibility (CSR) vehicles], think‐tanks and charities, with the alcohol companies themselves not being particularly visible [16, 17, 18, 19].

The key global policy context for TRAPS was the recognition by the World Health Organization (WHO) of the need to accelerate action to reduce alcohol harms [20, 21]. The World Health Assembly approved plans leading to an intensification of action [20]. This followed recognition that alcohol industry interference in policymaking was thwarting progress by delaying the implementation of the most effective measures [21, 22].

The TRAPS research programme was located within the Department of Health Sciences at the University of York. TRAPS comprised a multi‐disciplinary team of public health and social scientists, with collaborators across the globe. It was funded by the Wellcome Trust, with support from the University of York. Between 2016 and 2022, the team conducted studies that investigated the relationship between the alcohol industry, public health science and policy.

In this paper, we provide an interpretive synthesis of the findings from the TRAPS research programme. We identify some key implications for future research on alcohol and show why research on the alcohol industry as a dedicated object of study is vital to the advancement of alcohol public health research.

## METHODS

From the outset, TRAPS aimed to provide an empirical foundation for developing an orientation within alcohol sciences towards the alcohol industry as an object of study. Work was thematically organized in a series of four linked strands: the nature of the industry itself; industry involvement in science (evidence production); industry involvement in policymaking; and industry activity at the science–policy interface (evidence use by industry actors and management of the use of evidence by other parties). The decision was made to build new conceptual frameworks as the work advanced. This meant avoiding affording primacy to any particular discipline. Relatedly, we eschewed scaffolding ideas upon the analysis of any other industry (e.g. tobacco or pharmaceuticals) to enhance our capacity to identify novel practices or characteristics rooted in the observed features of alcohol industry activities. The ethos of TRAPS was thus highly empirical, conceptually eclectic and transdisciplinary, and this is reflected in the nature of this synthesis.

Through an iterative process of careful analytical engagement, we produced a new interpretive synthesis [23] and identified major themes to be considered in research agenda setting. The data set comprised every formally accepted TRAPS study published as of March 2022. The review was not a simple summary of all existing research undertaken within TRAPS, although it provides a meta‐summary of a substantive body of work. Rather, it integrated TRAPS findings interpretively across its component studies [24]. As such, the analysis was not pre‐registered, so the results should be considered exploratory. We identified key contributions made by individual studies to the literature on alcohol policymaking and science and, as a platform for thinking about future research priorities, reflected on overall TRAPS progress, identifying areas where this has been limited.

## RESULTS

TRAPS began by undertaking a series of systematic reviews that summarized and evaluated the content of the peer‐reviewed literature on industry involvement in science, CSR and policymaking [26, 27, 28, 29]. This identified serious long‐standing concerns in the research community regarding the integrity of the scientific evidence base itself, despite which there was little formal study of industry funding as a source of bias or interference in the processes of undertaking research [26]. Moreover, industry actors were identified as seeking to shape the evidence informing policymaking by making instrumental interventions in evidence production and use in policymaking. This review also showed that a substantial minority in the research community held opposing views to the concerns raised by the majority about the scientific activities of the industry [26].

The existing literature on alcohol industry involvement in policymaking, although small, was the most developed methodologically and provided the strongest evidence across these systematic reviews. This found that industry actors sought to keep alcohol issues off high‐level policy agendas by framing the issues in ways that protected commercial interests and narrowed the focus of discussion when policy debates arose [27]. In tandem, alcohol industry actors invested substantially in building long‐term relationships with policymakers to shape policymaking norms, including the promotion of ‘partnerships’ between industry and government. Industry actors pragmatically used different organizational forms in particular contexts [27]. Such sophisticated strategies were developed over time and have largely been successful throughout the world, even in the face of setbacks, at delaying or even reversing policy initiatives to reduce alcohol harms [27].

A systematic review of alcohol industry’s CSR initiatives found that these were used to influence the framing of the nature of alcohol‐related issues in line with industry interests. There was no robust evidence that the industry’s preferred initiatives were effective in reducing harmful drinking [28]. An important study limitation was that CSR initiatives fused with marketing were excluded because marketing was not part of the TRAPS remit. This review may therefore have understated the significance of CSR in advancing industry interests. Finally, a systematic review examined the integration of business, CSR and political strategies in public health surveillance studies of alcohol industry actors [29]. This found a high degree of collaboration in political strategy development between companies, facilitated by changes in the structure of the international alcohol industry and the growing concentration of global producers operating in increasingly oligopolistic markets [29].

### Alcohol policymaking studies

Minimum unit pricing (MUP) has been the key alcohol policy dispute in Britain during the life of the programme (see Box 1). MUP was first discussed in Scotland in 2008 and implemented after a long delay in 2018. The UK government announced its intention to implement MUP in 2012 but then reversed these plans. In 2017, the Welsh government legislated for MUP and implemented the measure in 2020. In England, we examined how ideas concerning partnership were institutionalized to limit the scope for MUP and other alcohol policy innovations [30]. A multiple streams approach demonstrated how a policy window opened for MUP and was then closed [31]. The backdrop was the longer‐running effort to adopt MUP in Scotland; there we examined industry strategies that successfully delayed implementation, including early and ongoing threats of the prospect of litigation [32]. These strategies included seizing numerous opportunities to block policies within the European Union’s multi‐level system of governance [33]. In Scotland [32], as in England, in the revision of low‐risk drinking guidelines [34] partnership rhetoric was cast aside and an adversarial, even threatening, posture was adopted when the industry’s interests were compromised. Industry actors chose carefully which battles to fight, where and when [35, 36]. In some countries, the alcohol industry has been shown to be highly dependent upon a minority of heavier consumers for a large proportion of its revenue [37]. Policy measures aimed at reducing consumption among this group of drinkers, such as MUP, are defined by industry actors as a key threat to its interests.

Box 1 **The experience with Minimum Unit Pricing (MUP) in Britain.**

*What is it?* MUP is a mechanism that establishes a floor price for a dose of alcohol, with retailers prevented from selling below it. MUP seeks to drive down demand for low‐cost, high‐alcohol products through price increases.

*Who does MUP affect?* Alcohol retailers, particularly supermarkets and off‐license retailers, alcohol producers through these constraints and consumers that regularly purchase and drink low‐cost, high‐alcohol products.

*Where was MUP implemented?* Scotland introduced a minimum price of 50p per unit of alcohol on 1 May 2018. Wales adopted similar legislation on 2 March 2020. England earlier decided to adopt MUP and then decided not to implement it.

*What were the main arguments for policy change?* Pricing interventions have long been recognized as among the most effective tools for reducing alcohol‐related harm within society. Where easy access to cheap alcohol is a significant driver of harm, increasing the cost of alcohol holds particular appeal. In both Scotland and Wales, increasing taxes on alcohol was not possible due to limits on government decision‐making authority. MUP was instead promoted and adopted.

*What have been the key barriers to policy implementation?* MUP legislation was opposed by the alcohol industry in Scotland. Led by the Scotch Whisky Association, the industry challenged the legality of the legislation at Scottish, UK and European levels. The legal challenges delayed MUP’s implementation in Scotland by approximately 6 years. In England, well‐positioned industry lobbying was prominent in a reversal of the decision to implement MUP. Wales proceeded slowly and carefully in anticipation of industry opposition.

*Why does this matter?* Well‐resourced industry opposition can seek to maintain the *status quo* by keeping alcohol issues off policy agendas. When a government considers making evidence‐informed alcohol policy decisions, they have reason to expect opposition from industry, however modest the measures. In some circumstances, opposition will continue after the formal decision making appears to have been concluded, both when industry has influence within government and when it does not.

Drawing upon expertise in political science, TRAPS developed a framework that incorporated the intersection of interests, institutions and ideas to take forward research on how industry actors use lobbying, framing and institutional access in policymaking [38]. This required situating industry in relation to competing actors, particularly those involved in public health advocacy [39], and in specific institutional contexts [40, 41]. In Wales, as was the case earlier in Scotland, limited industry organization at the level of the devolved administration constrained the ability to impede MUP policy development [36]. Unlike in Scotland, the implementation of MUP was not directly delayed by a series of legal challenges by industry [36]. Instead, slow progress of the legislation in Wales was in part a consequence of governmental caution about the prospect of an industry challenge. However, in this case, industry efforts to prevent or delay MUP were muted, providing further evidence of ‘venue shopping’ in the context of multi‐level governance.

In Ireland, MUP was just one component of a comprehensive and world‐leading package of alcohol policy changes. As such, it was not the focus of industry contestation, and other aspects of the legislation dominated political debate [42]. As in Wales, accumulated effects of earlier policy failures helped foster the emergence of a consensus among the major political parties that alcohol needed to be dealt with as a public health issue [39, 43]. Both the legacy of policy failure and innovations in the political organization of the public health community had concomitant consequences for the tactics used by industry actors, but these adaptations had limited success in weakening the legislation [42, 44]. Tactics included using involvement in the policymaking process to obstruct particular provisions; coalition‐building and mobilizing proxies, with the major companies barely visible; and making use of extensive resources in commissioning lobbying [44]. As seen previously in Scotland, high‐level political leadership and cross‐party support were needed to secure the opportunity for policy change once the window of opportunity opened [42]. As a consequence of these studies, researchers now have examples of instances in which industry interests did not prevail in alcohol policy decision‐making, with lessons that may be transferable to both policy research and policy advocacy elsewhere. These findings can help researchers to understand more clearly the scope of (and limits to) the alcohol industry’s political power and can potentially be applied and/or adapted to the study of industry influence in other public health contexts.

### Alcohol industry involvement in science

A TRAPS bibliometric study, which assessed declared funding in articles in the Web of Science suite of databases, revealed that alcohol companies and related organizations are much more extensively involved in scientific research than previously understood [45]. A co‐authorship network analysis of systematic reviews on alcohol, cardiovascular disease and industry funding [46] found that the design of studies differed between authors with histories of industry funding and those who did not. It also found the presence of distinct industry‐linked subnetworks, and that all reviews with industry funding connections reported positive outcomes for low‐dose alcohol consumption, in contrast to mixed findings throughout the wider literature [46]. TRAPS conducted a detailed analysis of the controversy that arose in 2018 concerning the Moderate Alcohol and Cardiovascular Health (MACH) trial, which secured two‐thirds of its $100 million funding from the alcohol industry and was supported by the US National Institutes of Health [25]. The MACH trial was designed to investigate the possible cardioprotective effects of alcohol and was terminated due to institutional failings that led to a biased trial design [47]. The TRAPS study showed how the process of soliciting research funding from large alcohol companies had intrinsically biased the trial; for example, by being designed to avoid showing negative outcomes [47].

Our interview study conducted with researchers working on alcohol policy‐relevant topics, the majority of whom had worked with the alcohol industry, examined the perceived impact of receiving alcohol industry research funding. This revealed enduring effects of receiving industry funding early in careers, despite individual grants having ‘no strings attached’ [48]. Senior researchers, who had collaborated with SAOs to make their work more evidence‐based, generally discontinued and regretted that work [49]. For researchers who had chosen to avoid working with industry, the alcohol industry was nevertheless a ubiquitous presence in their scientific lives, not least through active industry surveillance of the alcohol research field [50]. Those who produced work that ran contrary to industry interests were subject to interventions, including intimidation [50]. Almost all interviewees viewed alcohol industry involvement in research as damaging to the field in various ways and, drawing upon their own experiences, would advise junior colleagues to avoid industry research funding [51].

As with the MACH trial [47], scientific and public controversies have been fruitful sites to uncover other features of industry involvement in science. A TRAPS study showed how interventions in peer‐reviewed journals by SAOs, which challenged research papers critical of alcohol industry organizations, functioned to foster controversy about new evidence and bolster their appearance as legitimate scientific actors [52]. Another TRAPS study showed how an anthropological report commissioned by a major alcohol company was used to influence public policy decision‐making on alcohol and violence—another challenging issue for industry [53]. This was after the report had been exposed in this journal as being largely devoid of meaningful scientific contribution [54].

Alcohol industry engagement with science became more intensive in the mid‐1990s as the major companies became global operators [55]; its earlier roots remain largely understudied. TRAPS research has attempted to rectify this using the tobacco industry documents archive in several studies [56, 57, 58]. It is well known that Hill+Knowlton, a public relations (PR) company, developed and managed the tobacco industry’s scientific programmes from the early 1950s onwards [59]. A TRAPS study found that Hill+Knowlton was working with the US distilled spirits industry before it began working with the tobacco industry and that the two industries worked closely together at key moments in the subsequent decades [57]. As with tobacco, at the core of the alcohol industry approach was funding research to advance what were explicitly conceived as PR goals [57]. Facing what they saw as an existential threat in the 1980s, the alcohol industry developed a global network of SAOs to counter national alcohol policies, a network that has expanded and that continues to play an important role [57]. At the heart of the PR message is the idea that it is individual (heavy) drinkers who are the problem and they, not the product itself, should be the subject of focused intervention, a narrative that was first established in the 1950s [57].

### The science–policy interface

The alcohol industry has good reason to fear that alcohol is viewed similarly to tobacco, and to hide its close connections: both sectors sell drugs that are toxic and addictive and manipulate the dose of the drug for commercial purposes [60, 61]. They also recruit users when young through major investments in marketing. Both industries derive their profits from unhealthy levels of consumption [61, 62], and the rationale for regulation is basically the same [61]. Arguably, the overall societal impacts of alcohol are similar to tobacco [62]. Box 2 presents key TRAPS data on the relationship between the alcohol and tobacco industries.

Box 2 **Observations on relationships between the alcohol and tobacco industries during the past 70 years.**

1. The major strategic threat that population health protection poses to business interests has been recognized by both to be highly similar and deemed prohibitionist in nature [58].
2. From the 1950s onwards, both used research funding to mould how key issues were defined, studied and thought about among scientists, the public and policymakers, with close connections in how the strategies were developed [57].
3. Alcohol problems were framed in individual rather than in population terms, and the cause of the problem was seen as lying in the consumer and not the product in ways with strong parallels, for example, to the use of genetics as an explanation for lung cancer [27].
4. At key moments the two industries have worked together in closely guarded ways; for example, in monitoring World Health Organization (WHO) and other international agencies in the mid‐1980s [57].
5. Tobacco interests had key roles in the formation of the global alcohol industry strategy in the mid‐1990s and in US national trade associations [58].
6. Inter‐relationships in ownership, control and strategic collaborations continue to this day [58].
7. Public health policy is not the only public policy target for collaborations between the two industries; for example, they have recently worked together in trying to influence science policy [63].
8. Although the WHO Framework Convention on Tobacco Control has sought to exclude the tobacco industry from public health policymaking, the alcohol industry enjoys close partnerships with national governments [30] and within the UN intergovernmental system.
9. Alcohol industry corporate social responsibility (CSR) innovations such as the proliferation of ‘social aspects’ organizations in recent decades have parallels with the use of front groups and astroturfing by tobacco [28].
10. At different junctures in political strategy development, tobacco has been led by alcohol and vice versa; the alcohol industry has not simply been manipulated by the tobacco industry, even if it appears that the latter has been more influential [64].
11. Much more is currently known about the tobacco industry than the alcohol industry, which should be considered when interpreting these observations [62].

Conflicts in alcohol policymaking are, in an important sense, really all about science—in particular, whether the population‐level evidence should inform the societal response. Remarkably, the norm has been that the science is largely ignored, in line with the industry PR game. There are signs, however, that the tide in alcohol policy may be turning, as exemplified by the studies undertaken in Britain and Ireland by TRAPS and others. As MUP and other national policy innovations are evaluated and societal and public health benefits are identified [65], diffusion to other countries may be anticipated. It is also to be expected that industry will devote the resources needed to oppose such developments, including by further undermining science through research funding and by targeting science policy [63]. Box 3 offers high‐level proposals for areas to be considered in research agenda‐setting based on our findings.

Box 3 **Major themes to be considered in research agenda‐setting.**

1. How are strategies developed in major alcohol companies? For example, what are the specific mechanisms linking the tobacco and alcohol industries at strategic levels, including the roles of scientists and senior executive relationships and cross‐sectoral movements, both contemporaneously and historically?
2. What lessons can be drawn from the experience with the tobacco industry? How can these insights be used to inform more in‐depth study of the political economy, marketing and related activities of the major alcohol companies (and related corporate sectors)?
3. There are few countries in which studies of alcohol policymaking have been conducted. Retrospective studies can make use of public domain documents and key informant interviews to elucidate the roles of ideas and institutional characteristics.
4. Particular attention is warranted to low‐ and middle‐income countries where the alcohol industry is expanding, and to global institutions. There are no studies with analytical foci that are inherently cross‐national in nature, such as the receptivity of policy actors to ideas on alcohol policy options or the malleability of institutional features to industry influence.
5. Policymaking studies also need to be undertaken prospectively as policy debates unfold, so that research may contribute evidence that can be acted upon in a timely manner; for example, in countering misinformation in the guise of corporate social responsibility (CSR).
6. There is a need to recover alcohol science from the consequences of the long‐term project of the alcohol industry. In developing ideas for future directions, we need to develop an appreciation of the nature and magnitude of problems that are deep‐rooted.
7. Public health should give more prominent consideration to alcohol and its commercial determinants, and how it can contribute towards the renewal of the alcohol research field.
8. Society needs to determine what it wants from alcohol research and how it should be funded.

## DISCUSSION

TRAPS has made substantial contributions to the research literature on the alcohol industry, science and policy. This has come at a time when research attention has just begun to focus on alcohol policy, marketing and CSR, more so than on alcohol science itself (although see, e.g. [66, 67]). Wider attention is also being given to transnational corporations in the commercial determinants of health agenda [68]. The key contribution TRAPS makes is to demonstrate how essential a transdisciplinary approach is to understanding the nature of corporate political activity; the crucial role industry involvement in science plays in the development of corporate political power; and how public health actors have successfully overcome industry opposition to evidence‐based policies. TRAPS was built on modest scientific foundations, so fulfilling a research agenda‐setting function may be its most important longer‐term contribution.

The tobacco industry documents archive has enabled the beginnings of a historically grounded understanding of the contemporary practices of the alcohol industry [56, 57, 58]. This research has revealed how the alcohol industry has been actively involved in shaping alcohol science since its contemporary foundations more than half a century ago. However, the full extent to which that intervention has biased the research agendas of alcohol science, both historically and contemporarily, is not known. TRAPS may have located the tip of the iceberg. More fine‐grained attention to the roles of scientific and other forms of evidence in policymaking will also help to further expose the interests at play.

The political power of the alcohol industry is now much more clearly understood in the research literature, and recognition of the illusory nature of its key arguments has paved the way for the policy changes we have seen to date. It is important to give attention to two key limitations of the TRAPS programme: first, the lack of focus on low‐ and middle‐income countries, which is a limitation of the existing literature as a whole, and secondly, the lack of inclusion of the study of marketing, for which the research literature has expanded enormously in recent years and overlaps considerably with CSR (e.g. [69, 70, 71, 72]). These limitations are not separate, however, suggesting the importance of studying the fusion of political, CSR and marketing strategies and how they shape media and public understanding. For instance, industry making ‘knowledge claims’ about alcohol and alcohol harms outside scientific fora is increasingly well‐recognized within the research community [69, 73, 74, 75, 76, 77].

There are further limitations of this paper to consider. The aspiration here is to briefly appraise the contribution of the component studies within the context of the programme as a whole, and the understanding of the alcohol industry this permits. We have also reflected on the boundaries of this endeavour in constructing the synthesis, and this has afforded us the space to consider future horizons. The interpretive synthesis is substantive content rather than methodology focused, and as such does not critically evaluate the methodological quality of the TRAPS studies themselves. Studies are also interpreted and integrated only within the TRAPS programme, not across the wider literature. This review does not systematically explore how the alcohol literature has developed since the initial stages of TRAPS. Relevant studies in the years following the publication of our systematic reviews are not included, nor are older studies that were excluded from these by their study designs.

The original ambition of TRAPS was to help to define the alcohol industry as an object of study, and this has been fulfilled to the extent of identifying implications in seemingly distant parts of the alcohol research literature [78]. In various respects, all research on alcohol consumption and its consequences, including policies for responding to such impacts, is alcohol industry research. The actions of the industry producing, marketing and selling this commodity will have some influence on what is being studied [79]. Therefore, to a greater or lesser extent, attention should be paid to industry within the analysis.

We have begun to consider the possibilities for embracing the investigation of alcohol marketing within the kind of frame articulated here [80]. As corporate communications strategies seek to thoroughly integrate consumer marketing with CSR content and political strategies, alcohol advertising has political functions and may also subtly bias scientific thinking [75, 80]. In common with other key technological developments in contemporary capitalism, the goal is to persuade people without them being aware that their thinking and actions are being scrutinized and influenced [80]. Claims to be operating in the public interest serve to undermine rather than promote the professed causes, thus conforming to recent definitions of propaganda [81].

Big thinking and ambitious cross‐national research investments [82] are now required to further develop the science base. Importantly, cross‐industry studies are essential for building findings regarding the alcohol, tobacco and other harmful consumption industries’ science and policy shared ‘playbook’. This may permit more substantial benefits to accrue as high‐quality science informs the societal response. The scientific evidence production processes that inform policy developments may thus become mutually reinforcing, where they help policymakers to enhance the integrity and effectiveness of policymaking in the public interest [79]. However, we know that industry intervenes and influences science and its contribution to policy at multiple levels, and is increasingly well resourced to do so.

The research implications clearly extend far beyond a narrow focus on the industry itself. Beyond the conduct of specific research studies, there is a need to engage with the public and with policymakers. For reasons that should now be clear, both are extraordinarily complex undertakings, which require long‐term commitments. The situation in which we now find ourselves could well become worse unless we embrace the many challenges that are now more clearly identifiable than when TRAPS began.

The alcohol industry’s ‘scientific’ alcohol policy arguments are a PR sham; this emperor can now be seen to be wearing no clothes. The largest alcohol companies have globally attempted to—and have often been successful at—thwarting social and political responses to alcohol and the damage to health and welfare it causes. These successes have occurred, in part, by penetrating the institutions of alcohol policymaking where they exist or preventing them from being developed. The possible effects on science funding are important to consider.

The alcohol industry cannot be regarded in simplistic terms merely as a bad actor, but as a powerful set of corporate forces acting in their own interests that continue to do enormous damage to population health and society. Just as the industry has been strengthened by consolidation into a small number of global companies [83], and the problems that are a consequence of its commercial activities have grown, so too has the willingness of some national governments to use existing evidence to take action to protect population health and wellbeing. The challenges posed by globalized marketing cannot be managed at the national level alone, and hence have been a key priority for WHO [20, 21]. Policymaking will be helped by science that is clear about three things: first, the nature of the industry and its practices, including its relationships with the tobacco industry (it would be a grave error to ignore the long‐standing inter‐dependencies between these industries); secondly, the identification of false claims and distracting ideas perpetuated by the industry; and finally, the importance of exposing attempts to shape science and policy in the interests of powerful actors.

The thematic research agenda‐setting material generated here is not, in itself, prescriptive in nature. Rather, it draws attention to areas that can be seen as requiring development based upon the findings of the TRAPS research programme. These are directions in research that have been slow to develop, due in part to industry involvement in science, and in the shadow it casts over alcohol research, and also for many other reasons. It is for the alcohol research community and its stakeholders to reconsider history, present circumstances and possible futures.

## DECLARATION OF INTERESTS

There are no competing interests to declare.

## AUTHOR CONTRIBUTIONS

**Gemma Mitchell:** Conceptualization; investigation; methodology; project administration. **Andreas Filippou:** Conceptualization; investigation; methodology. **Su Golder:** Conceptualization; investigation; methodology. **Jack Garry:** Conceptualization; investigation; methodology. **Andrew Bartlett:** Conceptualization; investigation; methodology. **Mary Madden:** Conceptualization; investigation; methodology. **Jim McCambridge:** Leadership of all aspects of the programme including funding acquisition, conceptualization, investigation, methodology, project administration, writing. **Matthew Lesch:** Conceptualization; investigation; methodology; project administration.
